# Combating Obesity With Thermogenic Fat: Current Challenges and Advancements

**DOI:** 10.3389/fendo.2020.00185

**Published:** 2020-04-15

**Authors:** Ruping Pan, Xiaohua Zhu, Pema Maretich, Yong Chen

**Affiliations:** ^1^Department of Nuclear Medicine, Tongji Hospital, Tongji Medical College, Huazhong University of Science and Technology, Wuhan, China; ^2^Department of Biology, Massachusetts Institute of Technology, Cambridge, MA, United States; ^3^Department of Endocrinology, Internal Medicine, Tongji Hospital, Tongji Medical College, Huazhong University of Science and Technology, Wuhan, China

**Keywords:** obesity, brown fat, beige fat, thermogenesis, β-adrenergic signaling, UCP1, calcium cycling, glycolytic beige fat

## Abstract

Brown fat and beige fat are known as thermogenic fat due to their contribution to non-shivering thermogenesis in mammals following cold stimulation. Beige fat is unique due to its origin and its development in white fat. Subsequently, both brown fat and beige fat have become viable targets to combat obesity. Over the last few decades, most therapeutic strategies have been focused on the canonical pathway of thermogenic fat activation via the β3-adrenergic receptor (AR). Notwithstanding, administering β3-AR agonists often leads to side effects including hypertension and particularly cardiovascular disease. It is thus imperative to search for alternative therapeutic approaches to combat obesity. In this review, we discuss the current challenges in the field with respect to stimulating brown/beige fat thermogenesis. Additionally, we include a summary of other newly discovered pathways, including non-AR signaling- and non-UCP1-dependent mechanisms, which could be potential targets for the treatment of obesity and its related metabolic diseases.

## Introduction

In recent years, obesity has become an ever-growing public health crisis. Its related diseases include type 2 diabetes, hypertension, cardiovascular disease, and cancer. The treatments for obesity have been shown to be minimally effective and often come with a slew of side effects. Generally, the production of heat is accompanied by a concomitant increase in the lipolysis of triglycerides and the oxidation of fatty acids (1). Thus, stimulating thermogenesis is a useful tool with which to combat obesity. In addition to shivering thermogenesis, non-shivering thermogenesis plays an important role in energy homeostasis. It was originally thought to occur only in newborn humans as a means to maintain their body temperatures as there exists abundant brown fat in their body. However, in 2007, brown fat was discovered in adult humans using 18^F^-fluorodeoxyglucose positron emission tomography/computed tomography (18^F^FDG-PET/CT)-based imaging (2). Importantly, the activity of brown fat in humans is negatively correlated to body mass index (BMI) and positively correlated to glucose tolerance as well as insulin sensitivity (3). Thus, non-shivering thermogenesis has become an area of interest as a means to promote more robust basal metabolism and consequently reduce the prevalence of diseases caused by a surplus of energy stores.

Canonically, the metabolic effect of brown fat is mediated by the activation of β-adrenergic signaling and the regulatory effect of uncoupling protein 1 (UCP1). The former is mediated by norepinephrine which is released from the sympathetic nerve terminals, and the latter contributes to the generation of heat through the mitochondria (4). As a result, most efforts to induce brown fat thermogenesis in mammals have focused on developing β3-adrenergic receptor (AR) agonists. However, β3-AR is not specific to adipose tissue, and its global activation oftentimes leads to deleterious side effects. For this reason, recent efforts in the field have focused on better understanding the mechanisms of brown fat activation that bypass ARs.

## Brown/Beige Adipose Tissue Biology

### Brown/Beige Adipose Tissue

In a healthy adult human, as much as 20–35% of the body weight is composed of white adipose tissue (WAT) (5), located predominantly in the subcutaneous and the visceral regions of the body. However, during disease states such as obesity, BMI can be above 30 kg/m^2^. WAT serves as the main energy store for the body, while brown adipose tissue (BAT) dissipates energy into heat via non-shivering thermogenesis (6–8).

In humans, BAT is located primarily in the cervical, supra-clavicular, supra-adrenal, and para-spinal regions (2). Morphologically, brown adipocytes are composed of multilocular small droplets and abundant mitochondria, which play a crucial role in non-shivering thermogenesis. BAT innervation by the sympathetic nervous system is important for its development and activation (9). Classically, following cold exposure, norepinephrine is released from the sympathetic nervous system. It then binds to the β3-AR in brown adipocytes, leading to an activation of adenylyl cyclase, an increase in cAMP levels, and the activation of protein kinase A (PKA). This, in turn, induces lipolysis in brown adipocytes. Moreover, UCP1, a mitochondrial membrane protein expressed primarily in BAT, has been shown to play a key role in the process of non-shivering thermogenesis. It uncouples the respiratory chain of oxidative phosphorylation within the mitochondria, leading to a production of transmembrane proton flow and generation of heat. Prolonged β3-adrenergic stimulation has been demonstrated to be necessary for sustained thermogenic activity (10).

Beige adipocytes were defined by the Spiegelman group in 2012 (11). However, brown-like adipocytes in mice was described as early as 1984 by Young et al. (12). The cells were found to be distributed in WAT after cold exposure or adrenergic stimulation. Furthermore, beige adipocytes appear morphologically similar to brown adipocytes, express UCP1, and also generate heat in the form of non-shivering thermogenesis (13, 14). They are innervated by the sympathetic nervous system as well (14). Indeed the density of noradrenergic fibers dramatically increases in murine WAT depot after cold stimulation or transgenic overexpression of protein PR domain containing 16 (PRDM16), which is a main regulator of brown adipogenesis (15). This indicates the importance of sympathetic stimulus in the development of beige adipocytes. The presence of beige adipocytes in humans is supported not only by 18^F^FDG-PET/CT imaging but also by anatomical and transcriptome profiling, revealing that the supra-clavicular region of 18^F^FDG-positive depots mainly consists of beige adipocytes (16), while the cervical region consists of classical brown adipocytes (17).

### Targeting Brown/Beige Fat Thermogenesis

While skeletal muscle-mediated shivering thermogenesis consumes a great deal of energy in cold, non-shivering thermogenesis contributes to energy expenditure even at low levels of cold stimulation. It has been shown that both BAT and skeletal muscle play a role in non-shivering thermogenesis (18, 19). Under mild cold conditions, UCP1-based thermogenesis in BAT and sarcolipin-based thermogenesis in skeletal muscle work synergistically. When either thermogenic processes is impaired, the other is upregulated to maintain temperature homeostasis in mice (20). However, the mechanism of this functional crosstalk between BAT and skeletal muscle remains unclear. Furthermore, during prolonged cold exposure, muscle shivering intensity decreases while BAT activity increases (21). This suggests a pivotal role of BAT in thermogenesis under thermal stress. Therefore, increasing BAT mass and activity by stimulating its development and adrenergic response can be strategies to combat obesity in mammals.

Crucially, scientists have discovered that classical brown adipocytes share a common progenitor with skeletal myocytes (22). It has been shown that PRDM16, peroxisome proliferator-activated receptor γ (PPARγ), and CCAAT/enhancer-binding protein β (C/EBPβ) are master regulators of brown adipogenesis. PRDM16 has been shown to control the switch between skeletal myoblasts and brown adipocytes (22). Moreover, it binds directly to PPARγ to stimulate brown adipogenesis. C/EBPβ has been shown to play a crucial role in BAT development as well (23), binding to PRDM16 and initiating the switch from myoblast to BAT differentiation (24). Additionally, data indicate that PRDM16 binds to many other regulatory factors including peroxisome proliferator-activated receptor γ-coactivator 1α (PGC1α), PGC1β, euchromatic histone-lysine N-methyltransferase 1 (EHMT1), C-terminal-binding proteins (CtBPs), and early B cell factor-2 (EBF2). It likely forms a complex with these factors to regulate brown/beige adipocyte development (25–28). Although active BAT has been detected by 18^F^FDG-PET/CT imaging in adult humans after cold stimulation, it has primarily been found in people who are young and lean, with a lower BMI (3). Numerous studies have indicated that BAT activity is inversely related to BMI (8, 29–31). This may also likely be attributed to the increase in cold insulation and the subsequent protection of heat loss associated with higher adiposity. This paradox presents a challenge in simply targeting BAT to treat obese patients.

Since beige fat in humans is gradually recognized (16), scientists have honed on inducing beige adipogenesis to combat a variety of metabolic disorders. Unlike white or classic brown adipocytes, the origin of beige adipocytes is extremely heterogenous. Beige adipocytes have been reported to be transdifferentiated from white adipocytes (32, 33) or directly differentiated from distinct progenitors including PDGFRα^+^ (34), mural (35, 36), or MyoD^+^ progenitors (37). Numerous studies indicate that UCP1, one of the main regulators of adaptive thermogenesis, contributes to beige fat development (38–40). Moreover, classical beige adipocytes are governed by PRDM16 as well (41, 42). Deacetylation of PRDM16 and PPARγ by sirtuin 1 (SIRT1) stabilizes the PRDM16/PPARγ complex, contributing to beige adipogenesis (39, 43). Alternatively, SIRT1 is activated and regulated by Ca^2+^/calmodulin-dependent protein kinase β (CaMKKβ) and AMP-activated protein kinase (AMPK) (44–46), the latter of which plays a role in fatty acid oxidation. Other positive regulators of beige adipogenesis include bone morphogenetic proteins (47) and fibroblast growth factor 21 (48).

For years, targeting the β-adrenergic signaling pathway has been the therapeutic strategy to induce beige adipogenesis and thereby combat obesity. A variety of natural compounds and clinical medications used for treating metabolic diseases, shown in Table 1, have been shown to induce beige fat development. Of note, irisin and berberine are two molecules which show stimulatory effects on beige fat and brown fat in humans (51, 55).

**Table 1:** Molecules promotional for brown and/or beige adipogenesis and their potential targets.

**Molecules**	**Potential targets**	**References**
Thiazolidinediones	SIRT1-PPARγ	(43) (41)
Melatonin	UCP1-PGC-1α	(49)
Berberine	AMPK-PGC-1α and PRDM16	(50) (51)
Green tea	AMPK	(52)
Menthol	UCP1	(53)
Irisin	p38 MAPK-ERK	(54) (55)
Ginsenoside	PPARγ and AMPK	(56) (57)
Retinoic acid	p38 MAPK	(58)
Resveratrol	AMPK	(59)
Fenofibrate	PPARα	(38)
Curcumin	β3-AR	(60) (61)
Capsaicin	SIRT1-PPARγ-PRDM16	(39)
Artepillin C	UCP1 and PRDM16	(62)
Bitter melon seed oil	Mitochondrial uncoupling	(63)
Omega-3 fatty acid	UCP1	(64) (65)
Butein	Prdm4	(66)
Catecholamines	β-AR and mTORC1	(67)
Eicosapentaenoic acid	AMPK, PGC-1α, PPARγ, PRDM16, and UCP1	(68)
Dietary luteolin	AMPK and PGC-1α	(69)
AICAR	AMPK	(70)
Farnesol	PPARγ, CEBPα, and AMPK	(71)
Cryptotanshinone	AMPK and p38 MAPK	(72)
Albiflorin	AMPK and PI3K/AKT	(73)
Trans-anethole	AMPK-SIRT1-PPARα-PGC-1α	(74)
Magnolol	AMPK, PPARγ, and PKA	(75)
Xanthohumol	AMPK	(76)
(-)-Epigallocatechin-3-gallate (EGCG)	AMPK	(77)
L-Rhamnose	β3 -AR, SIRT1, PKA, and p-38	(78)
Grape pomace extract	PKA, AMPK, p38, and ERK PGC-1α, PPARγ, PRDM16, and UCP1	(79)
Phytol	AMPK	(80)
Raspberry	AMPKα1	(81)
Nobiletin	AMPK and PKA	(82)
Medicarpin	AMPK	(83)
Olaparib	AMPK- SIRT1	(84)
Genistein	AMPK	(85)
Dietary sea buckthorn pomace	AMPK-PGC-1α-UCP1	(86)
Zeaxanthin	AMPKα1	(87)
Trans-cinnamic Acid	AMPK	(88)
Metformin	AMPK	(89)
6-Gingerol	AMPK	(90)
Dietary apple polyphenols	AMPKα	(91)

*AMPK, AMP-activated protein kinase; AR, adrenergic receptor; C/EBP, CCAAT/enhancer-binding protein; MAPK, mitogen-activated protein kinase; ERK, extracellular signal-regulated protein kinase; mTORC1, mammalian target of rapamycin complex 1; PI3K/AKT, phosphatidylinositol3kinase/protein kinase B; PPAR, peroxisome proliferator-activated receptor; PGC-1α, PPARγ coactivator-1α; Prdm, transcription factor positive regulatory domain; SIRT1, sirtuin 1; UCP, uncoupling protein*.

## Potential Anti-obesity Drugs and Their Safety

### Adrenergic Receptor Agonists

Adrenergic signaling, in particular β3-AR, is a well-established pathway for BAT activation and beige fat development in response to cold temperatures. Common selective β3-AR agonists and antagonists have been summarized in a 2011 review by Bhadada et al. (92). Several β3-AR agonists have been shown to induce thermogenesis (93, 94). However, β3-AR are distributed throughout the body, including in the central nervous system, myocardium, blood vessels, smooth gastrointestinal and skeletal muscles, gallbladder, urinary bladder, prostate, etc. (95). Potential binding of β3-AR agonists with receptors located elsewhere may cause unexpected side effects.

Currently, some β3-AR agonists including mirabegron, vibegron, ritobegron, and solabegron have been extensively investigated. Some have even been approved for clinical use to treat overactive bladders and urinary incontinence (96–98). Although mirabegron has been found to induce BAT activity as measured by 18^F^FDG-PET/CT (99), increase non-esterified fatty acids by up to 68%, and boost resting energy expenditure by up to 5.8% (100) in humans, no β3-AR agonists has been approved to treat metabolic disorders thus far. The most common off-target binding sites of β3-AR agonists are myocardium and blood vessels (92, 101–103). Notably, it has been indicated that β3-AR stimulation is related to heart failure because of the negative inotropic effect of β3-AR agonists (104, 105). Additionally, different agonists present inconsistent effects on blood vessels (92). Some cause vasodilation, which may give rise to tachycardia, while others promote vasoconstriction, which is associated with high blood pressure. These potentially fatal side effects make β3-AR agonists unsuitable stimulants for thermogenic activity in the clinic.

### PPARγ Receptor Agonists

PPAR receptors also play a critical role in regulating whole-body energy homeostasis. These receptors are abundantly expressed in adipose tissue, liver, and skeletal muscle, in addition to immune and gastrointestinal systems, and are known to regulate brown adipogenesis as well as glucose uptake and lipid biosynthesis in WAT (106, 107). PPARγ receptor agonists, such as troglitazone, rosiglitazone, and pioglitazone, have been applied to treat metabolic disorders and type 2 diabetes due to their insulin-sensitizing effects (108). However, due to side effects such as hepatotoxicity, myocardial infarction, bladder cancer, and heart failure, PPARγ receptor agonists have largely been withdrawn from the market (109). Although some PPARγ receptor agonists, such as pioglitazone, have been shown to cause weight gain in humans (110, 111), studies have indicated that rosiglitazone may induce beige fat development in mice through the activation of the SIRT1–PRDM16 pathway (41, 43). This suggests that PPARγ receptor agonists may be leveraged to combat obesity. Yet due to the potentially fatal side effects mentioned above, their clinical use remains problematic. Currently, several dual-acting PPARγ agonists have been synthesized. Promising studies have shown that certain PPARγ agonists may be beneficial in treating metabolic disorders with minimal off-target effects (112).

## Non-canonical Mechanisms Involved in Non-shivering Thermogenesis

**Figure 1:**
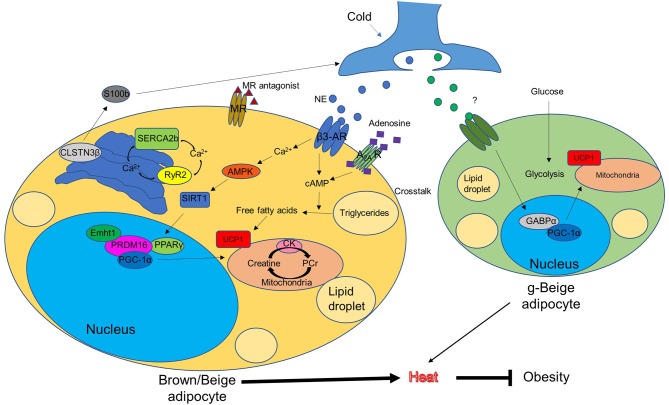
Conventional and unconventional mechanisms of brown/beige thermogenesis (potential approaches to combat obesity). AMPK, AMP-activated protein kinase; AR, adrenergic receptor; CK, creatine kinase; CLSTN3β, calsyntenin3β; Ehmt1, euchromatic histone-lysine N-methyltransferase 1; GABPα, GA-binding protein α; g-beige adipocyte, glycolytic-beige adipocyte; MR, mineralocorticoid receptor; NE, norepinephrine; PCr, phosphocreatine; PPARγ, peroxisome proliferator-activated receptor gamma; PGC-1α, PPARγ coactivator-1α; PRDM16, protein PR domain containing 16; RyR2, ryanodine receptor 2; SERCA2b, sarco/endoplasmic reticulum Ca^2+^-ATPase 2b; SIRT1, sirtuin 1; UCP1, uncoupling protein 1.

AR activation triggers the process of non-shivering thermogenesis in response to cold, as shown in Figure 1, while mitochondrial membrane protein UCP1 is the key driver of heat production in BAT. The UCP1 levels in beige fat are lower than in BAT. This has previously led to the misconception that the contribution of beige fat in the regulation of whole-body energy balance is marginal (113). However, UCP1 knockout mice without functional BAT can gradually adapt to and survive cold temperatures by increasing their recruitment of beige fat (114, 115). This suggests that UCP1 may be dispensable for beige fat induction. This phenotype suggests that other UCP1-independent mechanisms are involved in beige fat-regulated energy homeostasis. Furthermore, several studies have identified other pathways which activate BAT or induce beige adipogenesis, independent of ARs signaling (37, 116, 117). Here we describe a few novel mechanisms that have recently been implicated in the thermogenic regulation of BAT and beige fat (Figure 1).

### Adenosine–A_2A_ Receptor Signaling

A 2014 paper from the Pfeifer Lab describes adenosine–A_2A_ receptor signaling in response to sympathetic stimulation, which reduces levels of diet-induced obesity and improves glucose tolerance (116). After sympathetic stimulation by norepinephrine, brown adipocytes themselves release adenosine, which binds to A_2A_ receptors and contributes to energy expenditure. A_2A_ receptor knockout mice exhibit reduced thermogenesis and oxygen consumption in cold conditions compared to wild-type mice. Conversely, A_2A_ agonist treatment increases BAT activation and energy expenditure in mice. This highlights the important role of A_2A_ receptor in the regulation of energy expenditure in BAT. Furthermore, A2A stimulation by either its pharmacological activators or overexpression using lentiviral vector injections protects mice from diet-induced obesity while inducing beige fat development.

### Mineralocorticoid Receptor Antagonism

In mice, mineralocorticoid receptor antagonists prevent high fat diet-induced decline in glucose tolerance and induce beige fat development in visceral and inguinal WAT as indicated by an upregulation of brown adipocyte-specific transcripts and increased levels of UCP1. These findings correspond to the results detected by 18^F^FDG-PET/CT (117). Mineralocorticoid receptor antagonists reduce the autophagic rate in WAT depots. Moreover, when autophagy is repressed using its repressor bafilomycin A1, the effects mimic that of mineralocorticoid receptor antagonists. Furthermore, a more recent study in humans also indicates a positive correlation between mineralocorticoid receptor antagonism and BAT thermogenesis (118), suggesting the potential therapeutic benefit of mineralocorticoid receptor antagonism on obesity.

### Calsyntenin3β-S100b Signaling

A recent study from Spiegelman's group has identified a thermogenic adipocyte-specific protein [calsyntenin3β (CLSTN3β)], which is primarily located on the endoplasmic reticulum. This protein promotes sympathetic innervation in adipose tissue in mice (119). Knockout or transgenic overexpression of CLSTN3β in mice impairs or enhances sympathetic innervation in BAT, respectively. CLSTN3β activation leads to the secretion of S100b, a trophic factor which stimulates neurite outgrowth, from the thermogenic adipocytes. S100b deficiency reduces sympathetic innervation in BAT, while the forced expression of S100b rescues the phenotype caused by CLSTN3β ablation. Therefore, selectively targeting CLSTN3β-S100b in thermogenic adipocytes may minimize the off-target side effects in other organs and provide a new therapeutic opportunity for promoting thermogenic anti-obesity effects.

### Creatine-Driven Substrate Cycling

Another study from Spiegelman's group has identified arginine/creatine metabolism as a beige fat signature using quantitative mitochondrial proteomics (120). It contributes to beige fat-mediated energy expenditure and thermal homeostasis in mice. Cold exposure stimulates the activity of mitochondrial creatine kinase, which promotes creatine metabolism and in turn, increases ATP demand and induces ADP-dependent mitochondrial respiration in beige fat. Notably, in mice lacking UCP1, creatine metabolism compensatorily induces whole-body energy expenditure in response to cold. Furthermore, researchers identified phosphatase orphan 1 as a regulator of creatine-driven adipocyte respiration. It is concluded that creatine metabolism could be potentially targeted to increase basal energy expenditure.

### Sarco/Endoplasmic Reticulum Ca^2+^-ATPase 2b (SERCA2b)-Mediated Calcium Cycling

Another UCP1-independent signaling pathway in beige fat was described by our group. This novel mechanism involves sarco/endoplasmic reticulum Ca^2+^-ATPase 2b (SERCA2b)-mediated calcium cycling, which ultimately regulates glucose metabolism (121). Unlike brown adipocytes, beige adipocytes display higher ATP synthesis capacity. In the absence of UCP1, they gain fuel from glucose through multiple metabolic ways including glycolysis, TCA metabolism, and the mitochondrial electron transport chain through the SERCA2b-ryanodine receptor 2 (RyR2) pathway. Of note, the transgenic overexpression of PRDM16 is still able to protect mice from diet-induced obesity in the absence of UCP1. The present study strongly suggests that UCP1 is dispensable in beige fat for non-shivering thermogenesis. SERCA2b-mediated calcium cycling represents an evolutionarily conserved mechanism for maintaining energy homeostasis.

### Glycolytic Beige Fat

Our discovery of a distinct form of thermogenic cell was revolutionary in the field of fat biology. This cell, which was termed glycolytic beige adipocyte, exhibits adaptive thermogenesis and energy homeostasis in cold conditions in the absence of β-ARs signaling (37). These unique beige adipocytes are differentiated from MyoD^+^ progenitors in inguinal WAT. The process is mediated by GA-binding protein α through a myogenic intermediate. To better understand the mechanism by which these cells improved glucose tolerance and increased basal metabolism, we created a glycolytic beige fat-deficient mouse model. We found that glucose uptake, as detected by 18^F^FDG-PET/CT, in the inguinal WAT of those mice is significantly reduced. Moreover, we noticed a decrease in oxygen consumption rate and extracellular acidification rate in isolated tissues. Glycolytic beige adipocytes are distinct from conventional beige adipocytes in their developmental origin, regulation, and enhanced glucose oxidation. This β-AR-independent pathway has opened up a new path for the treatment of obesity.

## Discussion and Prospects

In mammals, brown fat and beige fat play a crucial role in non-shivering thermogenesis and energy homeostasis. Inducing their development or activation is a viable approach to combat obesity. Classic brown fat and beige fat thermogenesis is mediated by β3-AR signaling and UCP1. Previous research has focused on the development of β3-AR agonists or PPARγ agonists to treat metabolic disorders including obesity. However, the clinical outcomes are unsatisfactory due to their deleterious side effects. The added stress from these agonists to the cardiovascular systems is particularly harmful (103, 104, 108).

Alternative pathways which bypass canonical thermogenic regulators are of great interest. Surprisingly, UCP1 knockout mice and β-AR knockout mice are able to acclimate to cold environments (114, 115). This suggests that other compensatory pathways, independent of UCP1 or β-AR, are involved in regulating whole-body thermogenesis and energy homeostasis. Pathways associated with this acclimation, shown in Figure 1, include: two non-AR-dependent pathways mediated by other thermogenic cell-expressing receptors, such as A_2A_ receptors and mineralocorticoid receptors, whose activation by adenosine or inhibition by its antagonists contribute to energy expenditure; the thermogenic adipocyte-specific CLSTN3β-S100b signaling pathway, which regulates thermogenesis through promoting the sympathetic innervation of the thermogenic adipose tissue; two distinct UCP1-independent pathways in beige fat, including creatine-driven substrate cycling and SERCA2b-RyR2 signaling, which compensate for the loss of UCP1 and contribute to energy expenditure; and a subtype of beige fat, originating from MyoD^+^ progenitors, which is required for thermal regulation in the absence of β-ARs signaling.

It is important to note that these signaling pathways may only be a small part of the mechanisms involved in the regulation of BAT and beige fat on thermogenesis. Particularly, the role of beige fat in heat generation seems to be extremely multifaceted and, as such, is an active area of research. Notably, our group has identified glycolytic beige fat, marking for the first time that a subtype of beige fat has been described. We believe that multiple subtypes of beige fat with distinct origins and unique biological characterizations may exist. It is likely that there exists a robust crosstalk between different thermogenic cell types to maintain energy balance under different conditions. A better understanding of the plasticity of beige fat as well as of brown fat will likely provide new discoveries on metabolic adaptation and thus new therapeutic approaches to combat metabolic disorders including obesity.

## Author Contributions

RP and YC wrote the manuscript. RP, XZ, PM, and YC edited the manuscript.

### Conflict of Interest

The authors declare that the research was conducted in the absence of any commercial or financial relationships that could be construed as a potential conflict of interest. The handling editor declared a past co-authorship with the authors YC.
